# Editorial: Unraveling the complexities of premature aging: mechanisms and interventions

**DOI:** 10.3389/fragi.2026.1812422

**Published:** 2026-02-25

**Authors:** Mayank Choubey, Shivani Srivastava, Vinay Kumar

**Affiliations:** 1 Department of Foundations of Medicine, NYU Grossman Long Island School of Medicine, Mineola, NY, United States; 2 Department of Pathology, Yale School of Medicine, Yale University, New Haven, CT, United States; 3 Department of Medicine, Pennsylvania State University, Hershey Medical Center, Hershey, PA, United States

**Keywords:** accelerated aging, aging biomarkers, cellular senescence, genomic instability, inflammaging, premature aging, reproductive aging, telomere attrition

## 1 Introduction

This Research Topic in Frontiers in Aging brings together four contributions—both original research and review—that collectively examine premature aging from from mechanistic, clinical, and population-based angles. The articles focus on several hallmark processes of aging, including genomic instability, telomere shortening, chronic low-grade inflammation, and shifts in reproductive timing. By combining cellular and molecular studies with epidemiologic and genetic analyses, the Research Topic underscores how diverse, interacting pathways shape the onset and course of premature aging, as well as its potential reversibility.

Genomic instability is widely recognized as a central driver of premature aging, contributing to cellular dysfunction and tissue degeneration. In the first original research article, Chavan et al. investigated the reversal of perturbed DNA damage responses and HIV latency through pharmacological inhibition of histone methyltransferases in virally suppressed individuals living with HIV. The authors demonstrate that epigenetic modulation can restore defective DNA damage signaling pathways, thereby reducing viral persistence and aging-like genomic stress. These findings suggest that chronic viral infection and long-term antiretroviral therapy may accelerate biological aging through sustained epigenomic and genomic perturbations, and that targeting chromatin-modifying enzymes could represent a promising strategy to mitigate infection-associated premature aging phenotypes.

Telomere attrition represents another key hallmark linking premature aging to metabolic dysfunction. In a comprehensive review, Jinesh et al. synthesize current evidence connecting telomere shortening with metabolic diseases such as obesity, insulin resistance, and type 2 diabetes. The authors emphasize the bidirectional relationship between metabolic stress and telomere dynamics, wherein oxidative stress, inflammation, and impaired mitochondrial function accelerate telomere erosion, while telomere dysfunction further exacerbates metabolic decline. This review underscores telomere attrition as both a marker and mediator of premature aging, highlighting its potential utility in risk assessment and intervention strategies.

Chronic low-grade inflammation, commonly known as inflammaging, is widely acknowledged as a fundamental mechanism driving accelerated aging across various organ systems. In the third original research article, Zheng et al. analyzed data from the NHANES 2013–2018 cohorts to assess the association between systemic inflammatory indices and the risk of early natural menopause. Their findings reveal that elevated inflammatory markers are significantly associated with earlier reproductive aging, providing population-level evidence that inflammation contributes to accelerated endocrine decline. These results have important implications, as early menopause is associated with increased risks of cardiovascular disease, osteoporosis, and metabolic disorders later in life.

Reproductive timing also indicates early-life factors influencing long-term disease risk. Zhao et al. utilized a Mendelian randomization method in their fourth contribution to investigate the causal link between age at menarche and breast cancer risk in individuals and their first-degree relatives, categorized by estrogen receptor status. The study demonstrates that genetically determined earlier menarche is associated with an increased risk of breast cancer, reinforcing the concept that accelerated reproductive maturation represents an early-life manifestation of premature aging with long-term oncogenic consequences.

Collectively, the studies in this Research Topic illustrate that premature aging emerges from intersecting biological pathways involving genome maintenance, epigenetic regulation, telomere integrity, inflammatory burden, and endocrine control. Importantly, these contributions highlight that premature aging is not an irreversible process but rather a modifiable trajectory influenced by molecular, environmental, and lifestyle factors.

## 2 Future directions and perspectives

Future research on premature aging should emphasize integrative, longitudinal, and multi-omic methodologies to more effectively elucidate the dynamic interactions among genetic, epigenetic, metabolic, and environmental factors influencing aging. Single-cell and spatial omics technologies will be particularly beneficial for the identification of early molecular signatures of accelerated aging and the resolution of tissue-specific aging trajectories. In parallel, causal inference methods, including Mendelian randomization and systems biology modeling, will be essential for distinguishing drivers from consequences of premature aging.

From a translational perspective, interventions targeting epigenetic dysregulation, chronic inflammation, metabolic imbalance, and lifestyle-associated risk factors hold promise for delaying premature aging and extending healthspan. Ultimately, a deeper understanding of premature aging mechanisms will facilitate the development of precision aging strategies aimed at preserving physiological resilience and reducing early disease burden.

## 3 Conclusion

In conclusion, this Research Topic provides a comprehensive overview of current advances in premature aging research, spanning molecular mechanisms, population-based analyses, and genetic causal inference. By highlighting both shared and distinct pathways of accelerated aging, this Research Topic advances our understanding of premature aging and underscores the importance of integrative approaches for developing effective preventive and therapeutic strategies.

